# Engineering *Agrobacterium* for improved plant transformation

**DOI:** 10.1111/tpj.70015

**Published:** 2025-03-06

**Authors:** Greg S. Goralogia, Chris Willig, Steven H. Strauss

**Affiliations:** ^1^ Department of Forest Ecosystems and Society Oregon State University Corvallis Oregon 97331 USA

**Keywords:** genetic transformation, regeneration, transgenic, gene editing, CRISPR, recombineering, transposase

## Abstract

Outside of a few model systems and selected taxa, the insertion of transgenes and regeneration of modified plants are difficult or impossible. This is a major bottleneck both for biotechnology and scientific research with many important species. *Agrobacterium*‐mediated transformation (AMT) remains the most common approach to insert DNA into plant cells, and is also an important means to stimulate regeneration of organized tissues. However, the strains and transformation methods available today have been largely unchanged since the 1990s. New sources of *Agrobacterium* germplasm and associated genomic information are available for hundreds of wild strains in public repositories, providing new opportunities for research. Many of these strains contain novel gene variants or arrangements of genes in their T‐DNA, potentially providing new tools for strain enhancement. There are also several new techniques for *Agrobacterium* modification, including base editing, CRISPR‐associated transposases, and tailored recombineering, that make the process of domesticating wild strains more precise and efficient. We review the novel germplasm, genomic resources, and new methods available, which together should lead to a renaissance in *Agrobacterium* research and the generation of many new domesticated strains capable of promoting plant transformation and/or regeneration in diverse plant species.

## Significance Statement

Genetic transformation is a crucial tool in plant genetics research as well as a foundation for applying new plant breeding techniques. One of the bottlenecks to current plant transformation methods is the broad amenability of plants to *Agrobacterium*‐mediated gene delivery and promotion of organ regeneration. This review highlights recent advances in *Agrobacterium* genomics, novel tools to “engineer the engineer,” and ways that T‐DNA derived genes are being used to improve plant transformation and regeneration.

## INTRODUCTION

Genetic engineering, gene editing, and use of synthetic biology systems are potent tools for the modification of plant traits. However, these methods are difficult or impossible to accomplish in most species or genotypes aside from model systems (Altpeter et al., 2016; Atkins & Voytas, 2020). Although the insertion of transgenes is possible through various approaches, *Agrobacterium*‐mediated transformation (AMT) remains the most efficient, precise, and widely used method in public and private sector laboratories (Bélanger et al., 2024).

In the four decades since the first production of transgenic plants using AMT, we have learned a great deal about the biology and manipulation of these ubiquitous, gram‐negative soil bacteria (reviewed in Zupan et al., 2000; Gelvin, 2003; Nester, 2015). These include insights into the biological mechanisms of gall production, mechanisms of T‐DNA transfer, and how plant tissues provide nutrition. In brief, agrobacteria initiate crown gall or hairy root diseases by incorporation of transferred DNA (T‐DNA) into the host genome. T‐DNA transmission is coordinated by a gene cluster called the *vir* regulon that contains genes encoding, among other factors, components of the type IV secretion system (T4SS) (Gelvin, 2012). The T4SS functions as a bridge between bacterial and host membranes through which the T‐DNA is trafficked, along with the protein products of some *vir* genes. When the genes encoded on T‐DNA are expressed in plant cells, they induce gall or hairy root organ formation as a result of the expression of transgenes that alter plant hormone signaling (Gohlke & Deeken, 2014; Zhang et al., 2015). The T‐DNA also typically encodes one or more enzymes for the biosynthesis of unusual low‐molecular weight compounds called opines, which accumulate in the diseased organ and provide a nutrient source that agrobacteria are capable of utilizing as carbon and nitrogen sources (Vladimirov et al., 2015). It has also been established that *Agrobacterium* strains can be “disarmed” by the removal of phytohormone and opine biosynthetic genes present in T‐DNA, and yet still be highly effective in plant transformation. In addition, overexpression of *vir* genes and addition of *vir* inducers such as acetosyringone can increase strain virulence (van der Fits et al., 2000). Based on these biological insights and strain modifications, comprehensive systems for *in vitro* plant transformation have developed around a small set of disarmed strains produced in the 1980s and 1990s. These systems, though essential to generation of the majority of engineered and edited plant products to date, are inefficient or ineffective in the majority of plant taxa, particularly in dicotyledonous plants (Altpeter et al., 2016). Modern genomic and cloning tools should enable the generation of new strains that are more effective in a wide array of species. We review the growing genomic and genetic modification opportunities and provide suggestions for research and development.

## HISTORY OF *AGROBACTERIUM* STRAIN DOMESTICATION

**Figure 1:**
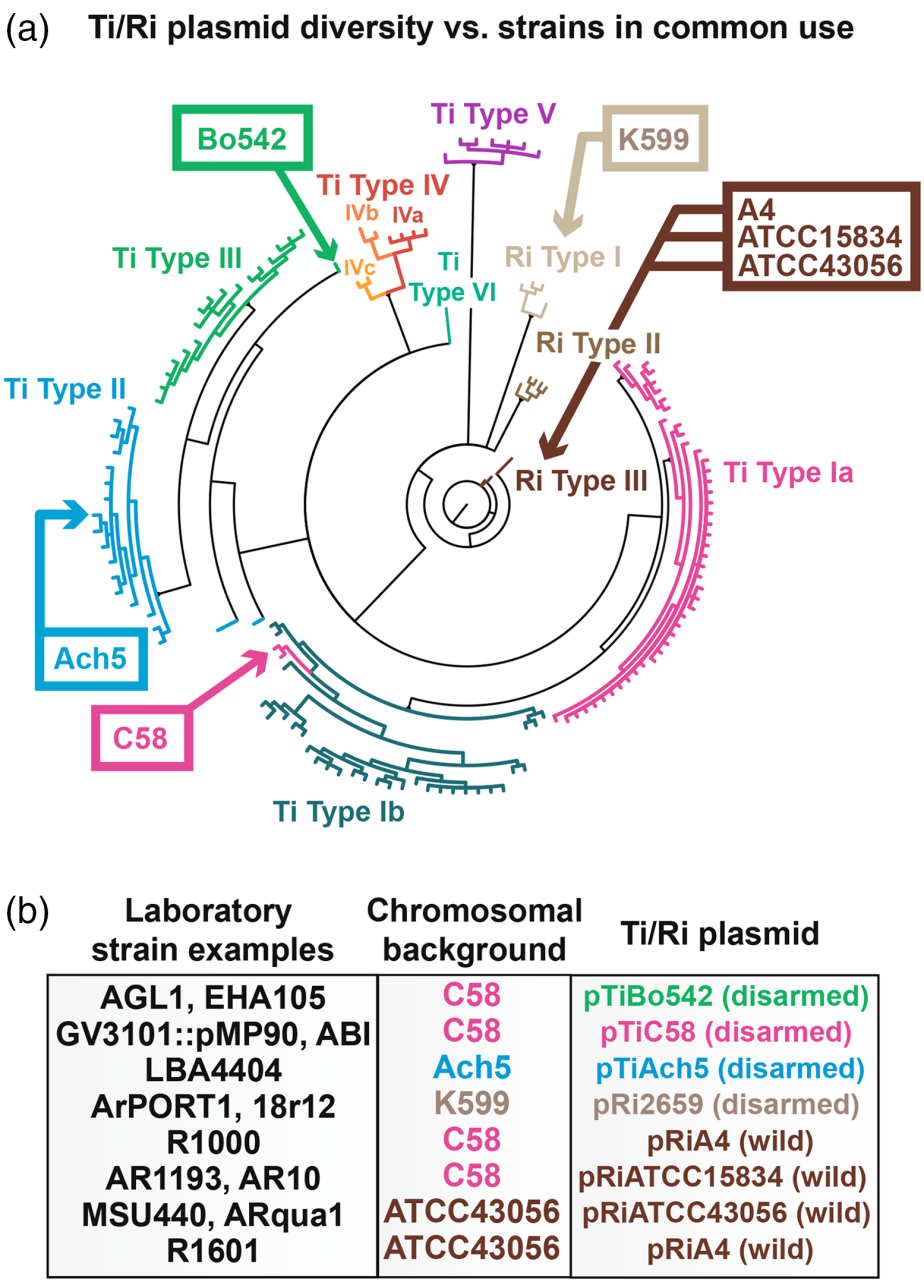
Phylogeny of sequenced *Agrobacterium* strain Ti and Ri plasmids illustrates the lack of diversity present among common laboratory strains. (a) Phylogeny of Ti and Ri plasmids reconstructed from Weisberg et al. (2020). Arrows indicate sources of strains or vir plasmids in common laboratory strains. Note that C58 and closely related Ti plasmids sometimes cluster within type Ib Ti plasmids rather than type Ia (their classified group) depending on the source genes used to assemble the tree (pers. comm.). (b) Examples of laboratory strain origin including the chromosomal background and the disarmed or Ri plasmid introduced into a given strain. Text colors in (b) correspond to the source clade from the Ti/Ri phylogeny in (a).

Extensive work took place in the 1980s to develop disarmed laboratory strains of *Agrobacterium* for use in plant transformation. Due to the techniques of the time, it took considerable effort to produce the disarmed strains in common use today. Many of the widely used laboratory strains have a C58 (type Ia) chromosomal background and may have a type III “hypervirulent” disarmed *vir* plasmid from strain Bo542 (such as EHA105 and AGL‐1), with the primary exceptions of LBA4404 (derived from type II Ach5), and partially disarmed versions of Chry5 (type III) (Figure 1a,b) (De Saeger et al., 2021; Weisberg et al., 2020). Ri plasmids have seen frequent use in hairy root transformation as “armed” wild strains or derivatives. Disarmed versions of Ri‐containing strains are available (using type I strain K599 as a background), but are less frequently used for routine transformation (Figure 1b) (Collier et al., 2018; Mankin et al., 2007). Mixing and matching many of these chromosomal backgrounds with armed or disarmed virulence plasmids has resulted in a set of strains available for laboratory use. However, these represent a very small proportion of the diversity of wild strains in public collections (Figure 1a,b) (De Saeger et al., 2021; Kiryushkin et al., 2022; Pennetti et al., 2024).

It is likely that the lack of diversity in laboratory strains limits plant transformation in many plant taxa, whether due to poor T‐DNA delivery or induction of host defenses. For example, testing of multiple strains of *Agrobacterium* in lettuce and tomato revealed very different profiles of transient GUS delivery and induction of necrosis responses (Wroblewski et al., 2005). Additionally, screening of strain collections for their ability to transform citrus tissues identified a novel strain with improved T‐DNA delivery and reduced explant necrosis across genotypes (Alabed et al., 2024). Complementation of mutants in *vir* machinery components (*virC*, *virD4*, *virD5*, and *virE3* genes) with versions from a variety of wild strains in laboratory strain GV3101(pMP90) showed slight improvements to transient T‐DNA delivery during agroinfiltration of *Nicotiana benthamiana* (Thompson et al., 2023). Complementation with the *virE* operon from pTiBo542 or pTiAch5 led to a substantial decrease in transient T‐DNA delivery in the same system, highlighting strain‐specific differences in *vir* gene potency for specific plants (Thompson et al., 2023). For protocol development of hairy root transformation and culture, there are often wide differences in transformation rate among strains, which suggest Ri plasmid‐type interactions with the plant species of interest (Kiryushkin et al., 2022). Thus, it appears likely that expanding the number and diversity of domesticated *Agrobacterium* strains will expand the efficiency and taxonomic range of effective AMT methods.

## MINING *AGROBACTERIUM* DIVERSITY WITH GENOMIC RESOURCES

DNA sequence information for *Agrobacterium*, particularly with respect to components of the T‐DNA transfer machinery or genes encoded on the T‐DNA, came slowly as efforts to characterize their biology, beginning in the 1970s, outpaced the development of sequencing technologies. The genome sequence of the widely studied strain C58 was first published in 2001. Since then, our knowledge of *Agrobacterium* genomics has increased many fold (Goodner et al., 2001; Wood et al., 2001). Modern sequencing technologies including short‐read and long‐read platforms have enabled assembly of many rhizobial genomes. There are currently over 350 unique strains from public collections around the world for which we have whole‐genome assembly data (with varying levels of resolution), as well as several hundred T‐DNA sequences from additional strains (Otten, 2021; Weisberg et al., 2020; Weisberg et al., 2021). Such sequencing efforts have provided great insight into the structure and evolution of these microbes and their unique lineages. These studies have also shown that pathogenic “agrobacteria,” as typically referred to by plant biotechnologists, are a paraphyletic group within the larger agrobacteria–rhizobia complex (ARC), for which extensive horizontal gene transfer is a hallmark of the origin and evolution of many of these species and strains (Weisberg et al., 2020).

The chromosomal backgrounds of sequenced genomes indicate that agrobacteria fall into three clades nested within a larger clade consisting of several other rhizobial genera (e.g., *Rhizobium*, *Neorhizobium*, *Ensifer*, *Shinella*) (Weisberg et al., 2020). Biovar 1 strains, which classically include *Agrobacterium tumefaciens*, can be further classified into eight “genomospecies” (i.e., a species differentiated from others using genotypic/genomic traits) (Weisberg et al., 2020). Biovar 2 strains, classically called *Agrobacterium rhizogenes*, have had a clear evolutionary bottleneck among extant strains compared to Biovar 1, with estimated species emergence in the last one to two million years. Biovar 3 strains, classically referred to as *Agrobacterium vitis*, form their own monophyletic group containing several genomospecies. In total, there are over 20 genomospecies level taxa among these groups (Weisberg et al., 2021). Genome architecture can differ considerably within agrobacteria. Most strains carry two discrete, multiple megabase‐length replicons, which are properly referred to as chromids since they possess properties of both chromosomes and plasmids (Harrison et al., 2010). Biovar 1 strains typically contain a larger circular chromid and a slightly shorter linear chromid, whereas biovars 2 and 3 contain two circular chromids, with each lineage having unique arrangements of gene clusters within each molecule (Slater et al., 2009). Strains may contain one to several non‐virulence accessory plasmids, and pathogenic strains carry one, or very rarely two, virulence plasmids (Weisberg et al., 2020). Overall, these genomic studies highlight the substantial chromosomal variation among strains in nature, which is poorly captured among disarmed laboratory strains.

There is evidence of extensive horizontal gene transfer of plasmids among agrobacteria, with virulence plasmid identity often poorly correlated with the chromosomal lineage. Encoded on plasmids is the machinery required to deliver DNA into plant cells (*vir* genes), one or multiple T‐DNAs, and other components such as those enabling opine metabolism (Nester, 2015). Virulence plasmids are typically labeled in classical fashion as tumor‐inducing (Ti) or root‐inducing (Ri) plasmids, based on their functionality *in planta*. They fall into at least 14 classifications among sequenced collections, with 3 Ri clades and 11 Ti clades (Weisberg et al., 2021). Among the Ti clades, some lineages are hybrid/mosaics of multiple plasmid types. Classically studied Ti plasmids for which T‐DNA or *vir* genes have been extensively analyzed in function include type Ia (pTiC58, pTiT37), type II (pTiAch5), and type III (pTiBo542), and typically contain one or two T‐DNAs (Figure 1a) (Weisberg et al., 2020). Ri plasmids typically deliver one or two T‐DNAs containing the root‐inducing *rol* genes, as well as a second T‐DNA carrying *iaaH* and *iaaM* auxin‐synthesizing genes in type III Ri plasmids (Figure 1a).

In addition to the identification of new *Agrobacterium* strains, another option aided by genomic data is to equip free‐living or endosymbiont rhizobia with the machinery necessary to deliver T‐DNA into plant cells. This may help to mitigate host defense responses during transformation as plants might be less likely to have strong immune responses to these generally non‐pathogenic bacteria. For example, this could include the transfer of a whole *vir* plasmid by conjugation into a recipient microbe, or a smaller “unitary” plasmid with a minimal *vir* gene cassette (but that is still able to confer T‐DNA transfer). Many examples of T‐DNA delivery in such strains have been shown, including for *Rhizobium trifolii*, *Phyllobacterium myrsinacearum*, *Sinorhizobium meliloti*, and *Mesorhizobium loti* (Broothaerts et al., 2005; Hooykaas et al., 1977). In the last decade, a similar approach has been shown for *Ensifer adhaerens* and *Ochrobactrum haywardense*, the former for rice and potato, and the latter for soybean (Cho et al., 2022; Rathore et al., 2015; Rathore et al., 2019; Wendt et al., 2012; Zuniga‐Soto et al., 2015; Zuniga‐Soto et al., 2019). Although the rate of T‐DNA delivery is significantly lower in transformation competent *Ensifer*, the innate immune responses are highly attenuated (Rathore et al., 2015). In *O. haywardense*, transformation of soybean was nearly twice that of two commonly used *Agrobacterium* strains, suggesting that such an approach will be most useful in species like soybean that have a strong necrotic response to *Agrobacterium* infection (Cho et al., 2022). However, learning to manage these poorly studied microbes can be challenging, with typical problems being antibiotic susceptibility and lack of amenability to liquid culture in common growth media without aggregation (Rathore et al., 2015).

## HISTORY OF *AGROBACTERIUM* MODIFICATIONS TO IMPROVE PLANT TRANSFORMATION

The types of modifications made to wild‐type strains in the early days of *Agrobacterium* research included removal of the virulence plasmid (i.e., “curing”), introduction of exogenous plasmids, and most importantly, removal of native pathogenic T‐DNA from a virulence plasmid while retaining T‐DNA transfer machinery (i.e., “disarmament”) (Barton et al., 1983; Fraley et al., 1985; Holsters et al., 1978; Hood et al., 1986; Hooykaas et al., 1977). Disarmament is a prerequisite for a strain to be useful in generating stable, normally growing transgenic crops. Early strain disarmament was usually facilitated by homologous recombination between a plasmid containing an antibiotic selectable marker flanked by digest fragments from the target virulence plasmid (called “homology arms”); the arms generally corresponded to sites that spanned some or all of the native T‐DNA region. The resulting plasmid, which typically lacked replication capacity in *Agrobacterium*, was then introduced into the target virulent strain via conjugal transfer. Homologous recombination between the two molecules occurs at a low rate, making the introduction of an antibiotic marker necessary to select for recombined colonies. However, resistant colonies must be further screened for susceptibility to another antibiotic marker present in the plasmid backbone to identify events in which double‐crossover homologous recombination has occurred, resulting in sequence replacement. This last step could be simplified by adding a *sacB* marker to the plasmid backbone (Gay et al., 1983), allowing counter selection against single‐crossover events, as retention of *sacB* causes colony death when grown on media containing sucrose (due to accumulation of the toxic product levan) (Mankin et al., 2007; Palanichelvam et al., 2000).

This same basic method of double‐crossover homologous recombination has been used to confer other genetic modifications to *Agrobacterium*. One of these is a knockout of the endogenous *recA* gene as was done in the development of laboratory strain AGL‐1 (Lazo et al., 1991). This mutation was intended to ensure against spontaneous recombination events in large cosmid vectors containing plant genomic fragments, and generally confers a degree of construct stability to binary plasmids (especially ones containing sequence repeats) when they are introduced into this background. However, the tradeoff for this mutation is that subsequent genomic modifications that rely on homologous recombination cannot be efficiently made in such *recA* ^*−*^ strains (Chen et al., 2008).

Auxotrophic mutants have been useful in making *Agrobacterium* easier to manage during transformation. By eliminating a strain's ability to synthesize an essential molecule, its growth can be more tightly controlled as the deficient molecule must be provided through media supplementation. Auxotrophic mutants are practically helpful during plant transformation as they largely eliminate the need for laborious and often damaging explant washes, and sometimes even the application of antibiotics. Homologous recombination has been used to knock out the *thyA* gene that encodes thymidylate synthase, resulting in thymidine auxotrophy (Ranch et al., 2010). It has also been used to knock out the *metA* gene that codes for homoserine O‐succinyltransferase, resulting in methionine auxotrophy (Prías‐Blanco et al., 2022).

Transposon insertional mutagenesis has been another tool used for *Agrobacterium* modification. The disarmed laboratory strain LBA4404 was derived from LBA4213, a Tn904 insertional mutant on the Ti plasmid of wild‐type strain Ach5 (Klapwijk Van Breukelen et al., 1980; Ooms et al., 1982). Several genes now known to be necessary for successful T‐DNA transfer to plants were mapped using mutagenesis libraries (Dale et al., 1993; Huang et al., 1990; Kang et al., 1992; Stachel & Nester, 1986). Tamzil et al. (2021) used this technique to generate a series of auxotrophic mutants for three different amino acids in an AGL‐1 strain background. However, this method is by necessity untargeted and requires laborious screening of many colonies to identify an insertion interrupting a particular gene of interest (Morton & Fuqua, 2012). Thus, as discussed next, when mutation targets are known, directed methods of mutation induction are becoming more common.

## NEWLY DEVELOPED TOOLS FOR ENGINEERING *AGROBACTERIUM* STRAINS

**Figure 2:**
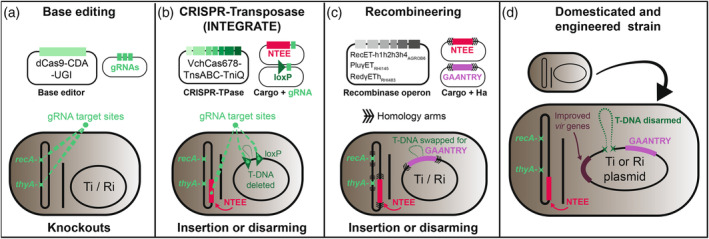
Gene editing and recombineering approaches to rapidly domesticate wild strains and enhance their utility in a laboratory setting. (a) Base editing to introduce premature stop codons into useful gene targets on the *Agrobacterium* chromosome. These include the *recA* gene to increase introduced construct stability, and the *thyA* gene to induce auxotrophy to eliminate the need to wash transformed explants and prevent overgrowth. (b) INTEGRATE system to insert cargo into specific sites in the *Agrobacterium* genome. This relies on a CRISPR‐associated transposase to insert cargo where directed to by a gRNA. For disarmament, lox sites are inserted flanking a native T‐DNA, then a *Cre* expression plasmid is added to induce excision of the T‐DNA. For insertion of other tools, gRNAs can be targeted to the chromosome or regions of the vir/Ti/Ri plasmid. INTEGRATE could also be used to disrupt genes with a cargo by inserting them into a gene coding sequence. (c) Recombinases isolated from rhizobia genomes and λRed systems can be used to induce homologous recombination with a native sequence. Cargo vectors include homology arms to the target site, usually also inserting an antibiotic resistance gene to improve recombinant identification. These could be used for simultaneous disarmament and insertion of sequences like a GAANTRY landing pad. This approach is most similar to traditional double crossover via triparental mating. (d) Example of a heavily engineered strain with ideal laboratory qualities. These include disarmed native T‐DNAs, increased construct stability, auxotrophy, delivery of non‐T‐DNA transformation enhancing elements (NTEEs) to reduce plant defense, GAANTRY construct assembly, and novel hypervirulent *vir* gene combinations.

Several technological developments have paved the way for far more expedient methods of strain engineering. These include PCR using high‐fidelity DNA polymerases, diverse low‐cost and widely available restriction enzymes, phage‐derived recombinase systems, *de novo* gene fragment synthesis capabilities, and the availability of “one‐pot” molecular assembly methods (e.g., Gateway, Golden Gate, and Gibson) (Engler et al., 2008; Engler et al., 2014; Gibson et al., 2009; Karimi et al., 2007). These have made recombinant plasmid construction far simpler than during the early stages of *Agrobacterium* modification. In addition, advancements in sequencing technology have enabled the generation of numerous, high quality, and publicly available genome assemblies and structural annotation data that allow straightforward targeting of nearly any candidate sequence. Directed evolution can be used to generate novel variants that enhance transformation rate; for example, changes to plasmid origins of replications led to increased binary plasmid copy number that enhanced T‐DNA transfer (Szarzanowicz et al., 2024). Finally, the discovery and engineering of CRISPR‐based systems for precise sequence alteration have led to the development of new tools for *Agrobacterium* genome modification that are not based on *recA*‐dependent homologous recombination. Some of the previously demonstrated and potential use cases for these novel tools are illustrated in Figure 2.

### GAANTRY

First described in Collier et al. (2018), GA*A*NTRY (Gene Assembly in *Agrobacterium* by Nucleic acid Transfer using Recombinase technologY) is a system that allows iterative *in vivo* construction of T‐DNA sequences for use in plant transformation. It uses two pairs of plasmids that do not replicate in *Agrobacterium* to facilitate stepwise incorporation of desired sequences at a pre‐existing site on the virulence plasmid. This cellular configuration obviates the need for binary plasmids, a widely used tool that was originally developed as a more convenient way of introducing T‐DNA destined for transfer to plants (as opposed to co‐integrant vector configurations) (Bevan, 1984; Hoekema et al., 1983). The complex stacking mechanism utilized in GA*A*NTRY relies on the actions of several site‐specific recombinase enzymes. As subsequent cargo stacking steps proceed, the T‐DNA alternates between carrying kanamycin or gentamicin resistance markers, allowing for theoretically unlimited rounds of stacking and the production of very long T‐DNA sequences. GA*A*NTRY constructs have been used to transform important crops including potato, rice, and soybean with large transgenes (Hathwaik et al., 2021; McCue et al., 2019; Shao et al., 2024).

The advantages of GA*A*NTRY assembly are that it allows for the incorporation of entire complex biochemical pathways or other large gene clusters into plants (whereas T‐DNA constructed in binary plasmids have a lower maximum size due to limited cargo capacity). Additionally, transgenic events produced show a high proportion of single‐copy insertion with low rates of sequences from outside the T‐DNA borders (Collier et al., 2018). One disadvantage, however, is that the GA*A*NTRY “landing pad” needs to be first installed onto the virulence plasmid before cargos can be stacked at that site. The backgrounds of the GA*A*NTRY‐compatible strains reported thus far may not be ideal for transformation in all plant species/genotype backgrounds. The landing pad was originally installed using double‐crossover homologous recombination. However, it should be feasible to use the other novel strain engineering tools described below to precisely insert this site onto disarmed virulence plasmids in new strain backgrounds.

### CRISPR/Cas base editing

Base editors are widely used to introduce targeted single‐nucleotide transitions rather than the insertions and deletions normally introduced by Cas9 and similar nucleases. Rodrigues et al. (2021) reported the development of a cytidine base‐editing system for use in *Agrobacterium* that was based on the Target‐AID architecture first demonstrated in *E. coli*. Target‐AID consists of a catalytically inactivated *Streptococcus pyogenes* Cas9 open reading frame fused to a cytidine deaminase (CDA1) and other elements (Banno et al., 2018). For use in *Agrobacterium*, this fusion was placed under the promoter sequence from *virB*, which had the intended effect of rendering base editor expression inducible by acetosyringone (AS). However, the authors reported leaky expression of the Target‐AID sequence, as successful base editing was observed even in the absence of AS induction. Thus, this step was ultimately excluded from the editing procedure.

In addition to the Target‐AID cassette, the base editing constructs used in this report consisted of sgRNAs for target genes expressed under a constitutive promoter, as well as the *sacB* gene under its native promoter, facilitating eventual plasmid eviction from the cell line following successful target editing. Base editing was demonstrated in strains EHA101, EHA105, and K599 with multiple genes targeted to generate early stop codons in each background. Two independently derived EHA105 variants mutant for *recA* were evaluated against the progenitor EHA105 strain for transient transformation in maize B104 immature embryos. There were no significant differences in plant reporter gene expression between the different strain treatments. Whole genome resequencing of multiple edited strains revealed off‐target base editing ranging from 17 to 60 sites, several of which fell within gene sequences resulting in non‐synonymous mutations (Rodrigues et al., 2021).

The components of this base editing system were made publicly available in a format that required the assembly of a final construct via multi‐site Gateway cloning. Pennetti et al. (2024) expanded on the original design of the base editing system by developing multiple vectors with varying antibiotic selection markers that avoid the need for Gateway assembly, reagents for which are cost‐prohibitive for many laboratories. These single‐component vectors are intended for direct cloning of sgRNA spacers and have been enhanced with chromoproteins, which serve as visual markers that aid in colony selection for plasmid eviction. In addition to generating *recA* ^−^ and *thyA* ^−^ variants of several strains commonly used in plant transformation (and in multiple newly disarmed strains), this group also developed a tool to filter sgRNA spacer candidates for generating in‐frame early stop codons in target genes.

### INTEGRATE–RNA‐guided sequence insertion

A system for targeted sequence insertion in *Agrobacterium* was reported by Aliu et al. (2022). The INTEGRATE system consists of a nuclease‐deficient type I‐F CRISPR/Cas system associated with a transposon (Tn6677) derived from *Vibrio cholerae* (Klompe et al., 2019). Complexes formed with these components can catalyze the insertion of a cargo sequence at a fixed distance downstream of a site determined by one or more crRNA spacer sequences. This system was first developed into an engineering tool and validated for making targeted insertions in *E. coli*, *Klebsiella oxytoca*, and *Pseudomonas putida* (Vo et al., 2021). The single‐component plasmid optimized for use in *Agrobacterium* consists of an operon containing the Cas/transposition machinery, single or multiple crRNAs, the donor DNA consisting of an mCherry expression cassette flanked by transposon left and right ends, and a *sacB* cassette to facilitate plasmid eviction. This construct design was used to generate *recA* ^−^ and *thyA* ^−^ variants of EHA101, EHA105, and AGL‐1 by disrupting the open reading frames through insertion of the mCherry cassette. The system's capacity for triplexed insertions was demonstrated in EHA105. An average of 42% of colonies screened were mutant for all three targets.

Long sequence deletions that resulted in the generation of disarmed variants was demonstrated for the two wild‐type strains C58 and Bo542 (Aliu et al., 2022). This was accomplished by a two‐step process that involved the insertion of a LoxP cargo at two sites on the Ti plasmid flanking the outermost left and right border repeats. To evict the INTEGRATE plasmid, a second plasmid was introduced that expressed Cre recombinase under a constitutive promoter. This new plasmid shared the same origin of replication (ORI) as the original INTEGRATE vector but contained a different antibiotic selection marker. This distinction ensured the successful eviction of the INTEGRATE vector when selecting for the Cre recombinase vector. Additionally, the dual LoxP sequences were recognized by Cre recombinase, leading to the successful excision of the intervening sequence between these sites from the virulence plasmid.

One limitation of this approach to disarmament is that cargos can be inserted in any orientation, which hinders subsequent sequence excision since both LoxP sites must be oriented in the same direction. Thus, sequencing based screening is required to identify colonies harboring LoxP insertions at both target sites with the same orientation before subsequent T‐DNA deletion. However, a major advantage of this method over double‐crossover homologous recombination is that disarmed variants can be readily obtained without the introduction of an antibiotic‐selectable marker. Retention of introduced antibiotic markers may limit compatibility with binary plasmids subsequently employed for plant transformation.

### Recombineering systems

Recombineering exploits bacteriophage‐derived non‐site‐specific recombinase systems (in contrast to site‐specific recombinases such as those employed in the GA*A*NTRY system). These systems can enable efficient sequence replacement of genomic targets, independent of the endogenous *recA* gene, through the introduction of double‐ or single‐stranded DNA templates containing relatively short segments at their ends which are homologous to the target sequence (Murphy, 2016). Prominent examples of bacterial recombineering systems are lambda Red and RecET. For an explanation of the molecular mechanism underlying recombineering using these systems, see Murphy (2016). Lambda Red and RecET both function well for the incorporation of linear fragments in *E. coli* and closely related bacteria, but are only marginally functional in more distantly related microbes, including *Agrobacterium* (Hu et al., 2014). Characterization of novel recombineering systems mined from phages or relics within sequenced microbial genomes has been previously used as a strategy to expand recombineering capabilities to new bacterial clades (Li et al., 2021; Wang et al., 2018; Yin et al., 2015).

Multiple novel systems for gene modification by recombineering in *Agrobacterium* were reported in Bian et al. (2022). Candidate recombineering operons were mined from publicly deposited *Agrobacterium* and *Rhizobium* genomic sequences using PSI‐BLAST searches with the RecT coding sequence as a query. Four different operons were identified and cloned in their entirety into parallel *Agrobacterium* expression vectors. To potentially boost the recombination efficiency of these native systems, additional constructs were made in which each operon was combined with either Redγ or Pluγ, genes which function in protecting the integrity of introduced double‐stranded DNA fragments.

Several inducible promoters were evaluated to enable tight regulation of these operons, after which 12 parallel constructs were generated, all based on the pBBR1 replicon, and each differing by unique recombineering operons expressed under a tetracycline promoter (intended for induction with anhydrotetracycline [AHT]). Each plasmid was tested for efficiency in inducing recombination between a linear fragment and target sequence in the backgrounds of strains C58, EHA105, and *A. rhizogenes* NBRC 13257. The same linear fragment was introduced into each strain using electroporation. It consisted of a resistance gene cassette for the antibiotic apramycin (Apra^R^) flanked by 80‐bp homology arms corresponding to sites on the recombinase expression plasmid. Recombinant events were selected by plating transformant cultures on media containing apramycin, and colony counts were used to compare the efficiency of each system. The recombination efficiency of the different constructs varied between the three strains. Thus, the researchers chose three of the systems, two of which incorporated the Pluγ protein, for advancement to subsequent experiments and to make publicly available. They subsequently optimized for each strain and recombineering system for several factors, including homology arm length, concentration of introduced linear DNA fragment, and conditions for cell preparation prior to electroporation. They also demonstrated efficient knockout of different target genes in each of the three strain backgrounds.

One limitation of these tools, at least in the plasmid formats they were made available, is the lack of a counter selection marker as are present in the base editor and INTEGRATE tools. Thus, these plasmids cannot be easily evicted from the strains following the desired genetic alterations. An advantage offered by recombineering over INTEGRATE for long sequence deletion/replacement (e.g., disarmament) is that it could potentially be completed in a single step with directional control of the inserted sequence. However, a drawback is that recombineering without insertion of an antibiotic selectable marker has yet to be demonstrated in agrobacteria.

## GENETIC ALTERATIONS TO IMPROVE T‐DNA TRANSFER

There are a variety of genes, gene variants, or gene clusters which, when expressed in *Agrobacterium* cells but not themselves transmitted to plant hosts, have been reported to increase rates of T‐DNA transfer or stable transgenic event production in some contexts. For the purpose of this review, these will be collectively referred to as non‐T‐DNA transformation enhancing elements (NTEEs). NTEEs can function by varying mechanisms, including directly facilitating more efficient T‐DNA transfer to host cells, modulating levels of plant signaling compounds, or either evading or mitigating plant pathogen defense responses normally triggered by *Agrobacterium*. Introduced NTEEs have been typically expressed from one or more accessory helper plasmids which co‐reside with a binary plasmid carrying a T‐DNA segment in the background of a strain containing the disarmed virulence plasmid. In several publications, this three‐plasmid cellular configuration has been referred to as a “ternary” vector system.

### Supplemental *vir* genes

One NTEE strategy that has long been understood as a way to boost plant transformation is the introduction of supplemental *vir* genes. *Agrobacterium* strains useful for plant transformation have been disarmed in such a way that they retain the full *vir* gene cluster present on the mutated Ti or Ri plasmid. About 20 genes in this cluster are essential for T‐DNA transfer (Nester, 2015). However, strong differences in virulence between strains have been documented, attributable to allelic variants of certain *vir* genes. There is also widespread variation between wild‐type virulence plasmids with respect to the presence/absence of non‐essential *vir* genes (Jin et al., 1987; Lacroix & Citovsky, 2022). One variant of note which confers high virulence is *virG*_N54D_, a so‐called “constitutive” mutant that mimics the phosphorylated state of the wild‐type *virG* allele that functions as a transcriptional activator of the entire *vir* regulon upon signal perception of plant‐derived phenolic compounds by *virA*. Ectopic expression of *virG*_N54D_ has improved plant transformation efficiencies in crops such as cotton, maize, and *Catharanthus roseus* (Hansen et al., 1994; van der Fits et al., 2000). A strategy developed by Japan Tobacco, Inc. utilized a plasmid with a cloned fragment containing *virG* along with *virB*, *virC*, and (truncated) *virD* operons, which was introduced into strain LBA4404 and designed to form a co‐integrate with a binary plasmid (Komari et al., 1996). This series of constructs that provided a partial supplement of *vir* genes were termed “superbinary” plasmids and assisted in elevating transformation rates in tobacco, rice, and maize (Hiei et al., 1994; Ishida et al., 1996; Komari et al., 1996). Later, ternary “helper” plasmids containing an expanded set of supplemental *vir* gene operons were developed, conferring significantly improved transformation, especially in monocots (Anand et al., 2018; Anand et al., 2019). Like the superbinary plasmids before them, the *vir* genes used to construct these helper plasmids originated from the so‐called “hypervirulent” type III Ti plasmid pTiBo542, disarmed versions of which are present in the strains EHA101, EHA105, and AGL‐1. They confer the highest improvement in transformation efficiency when added to the LBA4404 background, which carries a different (type II) Ti plasmid (Aliu et al., 2024; Kang et al., 2022; Zhang et al., 2019).

### Plant hormone‐modulating genes

Several studies have shown that plant hormones or other small signaling compounds can influence T‐DNA delivery to plants. The likely mechanisms include supporting polar attachment to cells within the host explant, interruption of the *Agrobacterium* quorum sensing signal, or inhibition of *vir* gene induction (Chevrot et al., 2006; Nonaka et al., 2008; Sardesai et al., 2013; Yuan et al., 2007). Thus, genes that modulate levels of these compounds have been employed as NTEEs. The *Agrobacterium* gene *tzs* (*trans*‐zeatin synthase) encodes a plant hormone‐synthesizing enzyme, which maps to the virulence plasmid outside of the T‐DNA region but is induced by acetosyringone (Beaty et al., 1986). It is only present on some Ti plasmids, including derivatives of C58. Hwang et al. (2010) showed that the presence of a functional *tzs* gene in an *Agrobacterium* strain can either aid or inhibit transformation depending on plant species/genotype.

The plant hormone ethylene is known to have a negative effect on *Agrobacterium vir* gene induction and gamma‐amino butyric acid (GABA) can disrupt bacterial quorum sensing (Chevrot et al., 2006). A research group at the University of Tsukuba in Japan has developed multiple iterations of plasmids expressing the bacteria‐derived genes *acdS* and *gabT*, which catalyze the degradation of ethylene's biosynthetic precursor and GABA, respectively (Nonaka et al., 2017; Nonaka & Ezura, 2014). Also, their most recent publication reported a 3.6‐fold enhancement to stable transformation in Micro‐Tom tomato using a plasmid expressing both *acdS* and *gabT* (Nonaka et al., 2019). Salicylic acid (SA) is a plant signaling compound associated with response to biotic stresses, but it can also directly inhibit *vir* gene expression in *Agrobacterium*. The bacteria‐derived gene *nahG* codes for an enzyme which can degrade SA to catechol. Anand et al. (2008) showed that tomato plants stably transformed with *nahG* are hypersusceptible to *Agrobacterium* infection, and Rosas‐Díaz et al. (2017) developed stably transgenic Arabidopsis lines into a tool for transient transformation studies. Jeong et al. (2024) recently detailed the construction of a ternary vector expressing *acdS*, *gabT*, *nahG*, and *virG*_N54D_, which conferred a 2.5‐fold increase in stable transformation in the Korean tomato cultivar “Hongkwang.”

### Genes/systems enabling evasion of host immune response

Plants have evolved mechanisms to recognize certain molecular signals, called pathogen‐associated molecular patterns (PAMPs), which trigger a rapid immune response known as PAMP‐triggered immunity (PTI). It involves the production of reactive oxygen species, reinforcement of plant cell walls by callose deposition, transcriptional activation of defense‐related genes, and stomatal closure limiting pathogen ingress (Zipfel & Robatzek, 2010). PAMPs tend to be molecular fragments which are common to entire classes of pathogens and are perceived by plant pattern recognition receptors (PRRs). Elongation factor Tu (EF‐Tu), an essential component of the bacterial translation machinery, is the primary PAMP known to trigger plant defense response to *Agrobacterium* and is perceived by the plant receptor EFR (Kunze et al., 2004; Zipfel et al., 2006). Yang et al. (2023) demonstrated a method for engineering PTI evasion in *Agrobacterium* by substituting the endogenous gene coding for EF‐Tu with an ortholog from *Pseudomonas syringae* pv. *tomato* DC3000, which contained divergent peptide residues and reduced induction of plant defense responses.

Another strategy to overcome PTI is through the transfer of “effectors” into host plant cells (Zipfel & Robatzek, 2010). Effectors are proteins that disrupt the processes inducing PTI by a variety of molecular mechanisms, including by direct interaction with plant PRRs and at downstream nodes in the signaling cascade (Zhang et al., 2022). Bacterial pathogens including *Pseudomonas*, *Xanthomonas*, and *Ralstonia* utilize effector proteins during host infection that are specifically transferred by a type III secretion system (T3SS). Although *Agrobacterium* species do not possess T3SS gene clusters, a landmark study by Raman et al. (2022) demonstrated that an entire T3SS gene cluster from *P. syringae* could be expressed heterologously in *Agrobacterium* strains. When used along with type III effector genes such as *AvrPto*, it led to substantial improvements in both transient T‐DNA transfer and stable transformation. This innovation was particularly noteworthy as 2.5‐ to 4‐fold enhancements in transformation were achieved in multiple dicot and monocot crop species, suggesting the possibility for wide application. Another surprising finding was that a plant‐derived protein previously shown to improve susceptibility to AMT in plant overexpression lines, H2A‐1, could be expressed in *Agrobacterium* with an N‐terminal type III secretion tag and transferred via the T3SS into plants, also leading to greater than fourfold improvements in transformation frequency (Tenea et al., 2009; Zheng et al., 2009).

### Improving *Agrobacterium* NTEEs

The typical constructs that have been built to express NTEEs in *Agrobacterium* are deployed as one or more accessory plasmids introduced into strains that also carry a disarmed Ti plasmid and a binary plasmid containing the T‐DNA. This configuration requires the co‐residing plasmids to have unique antibiotic resistance markers and to belong to different incompatibility groups, which could present constraints for future development of *Agrobacterium* strains. For example, it may be desirable to deploy different NTEE tools in combination, which would be difficult due to the limited number of broad host range plasmid replicons and antibiotic selection systems available that function well in *Agrobacterium*. One potential solution would be to produce strains in which NTEE modules are incorporated into the virulence plasmid, an endogenous accessory plasmid, or at a chromosomal locus. Some of the novel strain engineering tools discussed earlier could be deployed for this purpose. A possible candidate for chromosomal expression of NTEEs is the *pgl*/*picA* gene locus, for which it has been established that insertion at this position does not negatively influence plant transformation (Lee et al., 2001; Oltmanns et al., 2010; Rong et al., 1990).

Efforts to simultaneously utilize multiple NTEE modules would be greatly aided by the development of additional gene expression tools for *Agrobacterium*. Synthetic promoters from the Anderson collection (designed for expression in *E. coli*) are commonly used to provide constitutive expression in plasmid constructs. However, there has yet to be a systematic characterization of their activity in *Agrobacterium*. Several recent reports have detailed the development of some inducible and synthetic promoter systems for *Agrobacterium*, but broadening the toolkit of expression components could open further avenues for strain enhancement (Qian et al., 2021; Thompson et al., 2023; Toh et al., 2023).

Future development of NTEEs that reduce the degree to which *Agrobacterium* strains activate PTI could be useful. Following the model in Yang et al. (2023), other known PAMPs carried by *Agrobacterium* could be engineered to avoid triggering specific PRRs. Such molecular candidates could include lipopolysaccharide, peptidoglycan, and bacterial cold‐shock proteins (Erbs & Newman, 2012; Saur et al., 2016). For example, it has been shown that suppressing expression of the PRR recognizing cold‐shock protein *Nb*CORE in *N. benthamiana* led to a reduction in the plant immune response such that older plants could be efficiently agroinfiltrated (Dodds et al., 2023). This suggests that modification of the cold‐shock peptide sequence in *Agrobacterium* would be another route to achieve this same effect. However, the multiple copies of these genes in *Agrobacterium* would make accomplishing this more challenging than for the single‐copy EF‐Tu.

*Agrobacterium* strains expressing a T3SS also open up a plethora of possibilities for methods to suppress plant defense and boost T‐DNA transfer. Since type III effectors from a wide range of plant pathogens inhibit the PTI response through many distinct mechanisms, screening additional candidate effectors and combinatorial expression may further increase transformation efficiency and unlock unique transformation capabilities for individual recipient plant backgrounds. Additionally, the possibility of exploiting T3SS‐mediated protein transfer into plants provides many options for testing genes known or suspected to improve transformation which may be derived from plant, bacterial, or other sources, all without stably incorporating them into the target plant genome – an outcome which is undesirable as pleiotropic effects on plant growth or fertility are common. If complex NTEEs such as the *Pseudomonas* T3SS come into wider and more routine use for plant transformation, re‐engineering the gene cluster for reduced size and optimal expression of the individual components would likely provide further enhancements, similar to what was done with a T3SS cluster derived from *Salmonella* (Song et al., 2017).

## *AGROBACTERIUM* T‐DNA GENES TO IMPROVE PLANT REGENERATION

In addition to improving T‐DNA delivery, *Agrobacterium* genes can be used to improve plant regeneration, which is a large obstacle to plant transformation in many species (Bennur et al., 2024; Benson, 2000; Monthony et al., 2021). In nature, agrobacteria promote plant regeneration using T‐DNA genes that induce crown gall or hairy root disease. In conventional AMT approaches, regeneration is usually stimulated by the application of exogenous plant growth regulators (PGRs) such as phytohormones. The use of PGRs *in vitro* is a challenge, as extensive optimization is required to adapt the culturing procedures to new plant species and genotypes (Bélanger et al., 2024). There has been increasing interest in and use of morphogenic regulator genes encoded on construct T‐DNAs as a tool to improve plant regeneration responses, with major gains reported for transgenic event recovery in monocots, and to a lesser extent also in dicots (Gordon‐Kamm et al., 2019; Lowe et al., 2016; Wang et al., 2023). Many of the genes used in these configurations include plant meristem development factors such as *WUSCHEL*, *BABY BOOM*, and *GROWTH REGULATOR FACTOR4*/*5*‐*GRF‐INTERACTING FACTOR1* chimeras, but many other genes and combinations of them have been used in recent years (Debernardi et al., 2020; Duan et al., 2022; Hoerster et al., 2020; Lowe et al., 2016; Maher et al., 2020).

There is a long history of use of naturally encoded T‐DNA genes to improve plant transformation. Perhaps because genes encoded on the T‐DNA are under strong negative size selection to limit T‐DNA length, they have often evolved potent genes that interfere with natural host hormone and signaling pathways (Otten, 2021). Some of the most prominent are the cytokinin and auxin‐producing genes found in many Ti plasmids. *iaaH* and *iaaM* are two genes that influence auxin biosynthesis; together they act as two subsequent steps in a pathway leading to the formation of indole acetic acid (IAA) from tryptophan (Mashiguchi et al., 2019). Another gene, *ipt*, encodes a product that is localized to plastids and functions similarly to *iaaH/M* by catalyzing the reaction between 1‐hydroxy‐2‐methyl‐2‐(E)‐butenyl 4‐diphosphate (HMBDP) to trans‐zeatin and related cytokinins, bypassing endogenous cytokinin biosynthesis pathways in plants (Sakakibara et al., 2005). In the natural *Agrobacterium* pathogenesis process, *iaaH*/*M* and *ipt* form a positive feedback loop, trapping plant cells in a de‐differentiated state and maintaining gall proliferation (Zhang et al., 2015).

Early uses of these genes in transformation often included the presence of a recombinase‐excision cassette or transposase with flanking elements to enable the removal of the hormone‐producing genes after the initial regeneration of transgenic tissues. Such use cases included either *ipt* alone or the entire suite of *iaaH/iaaM* and *ipt* genes, and were found to be functional in multiple plant species, including tobacco, aspen, and rice (Ebinuma et al., 1997; Ebinuma et al., 2004; Ebinuma & Komamine, 2001; Endo et al., 2002; Matsunaga et al., 2002; Sugita et al., 2000). Other genes such as *6b* have been shown to have morphogen‐like effects on shoot proliferation *in vitro*, but the molecular functions of many T‐DNA‐encoded genes remain unknown (Otten, 2021; Wabiko & Minemura, 1996). In rare cases, some strains have been found which naturally induce the formation of “shooty” phenotypes and have been leveraged in wild strain co‐transformation frameworks similar to hairy root induction methods (Aronen et al., 2002; Azmi et al., 1997; Drevet et al., 1994; Marie‐France et al., 1990). Recently, there has been a resurgence in the use of the *ipt* gene as a morphogen for transformation, including in combination with plant meristem master‐regulator genes such as *WUSCHEL* and *SHOOT MERISTEMLESS* (Maher et al., 2020). The non‐cell autonomous nature of some of these *Agrobacterium* genes confers an advantage for specialized transformation systems such as tissue culture‐free transformation of greenhouse “*in planta*” materials.

Hairy root‐inducing *rol* genes on Ri plasmids also have strong development‐modifying properties. On most Ri T‐DNAs, this includes a well‐studied six‐gene region containing *rolA*, *rolB*, *rolC*, *rolD*, and the ORF13 and ORF14 genes between *rolC* and *rolD*, though other ORFs closer to the T‐DNA left border, such as ORF8, also contribute to hairy root production (Aoki & Syōno, 1999; Nilsson & Olsson, 1997; Ouartsi et al., 2004). Mutational studies have shown that the primary factor required for hairy root formation is RolB, and until recently its mechanism of action was unknown. Gryffroy et al. (2023) recently showed that RolB interacts with the TOPLESS co‐repressor proteins to interfere with auxin and jasmonic acid signaling, finally shedding light on this long‐held mystery. ORF13 and ORF14, though not essential for hairy root formation like *rolB*, enhance their formation rate (Aoki & Syōno, 1999). ORF13 has a retinoblastoma‐binding motif and can induce *KNOTTED1‐like homeobox* (*KNOX*) gene expression, which may increase meristem‐formation competency (Stieger et al., 2004).

### Innovations using *A. rhizogenes*‐mediated transformation in recalcitrant crops

**Figure 3:**
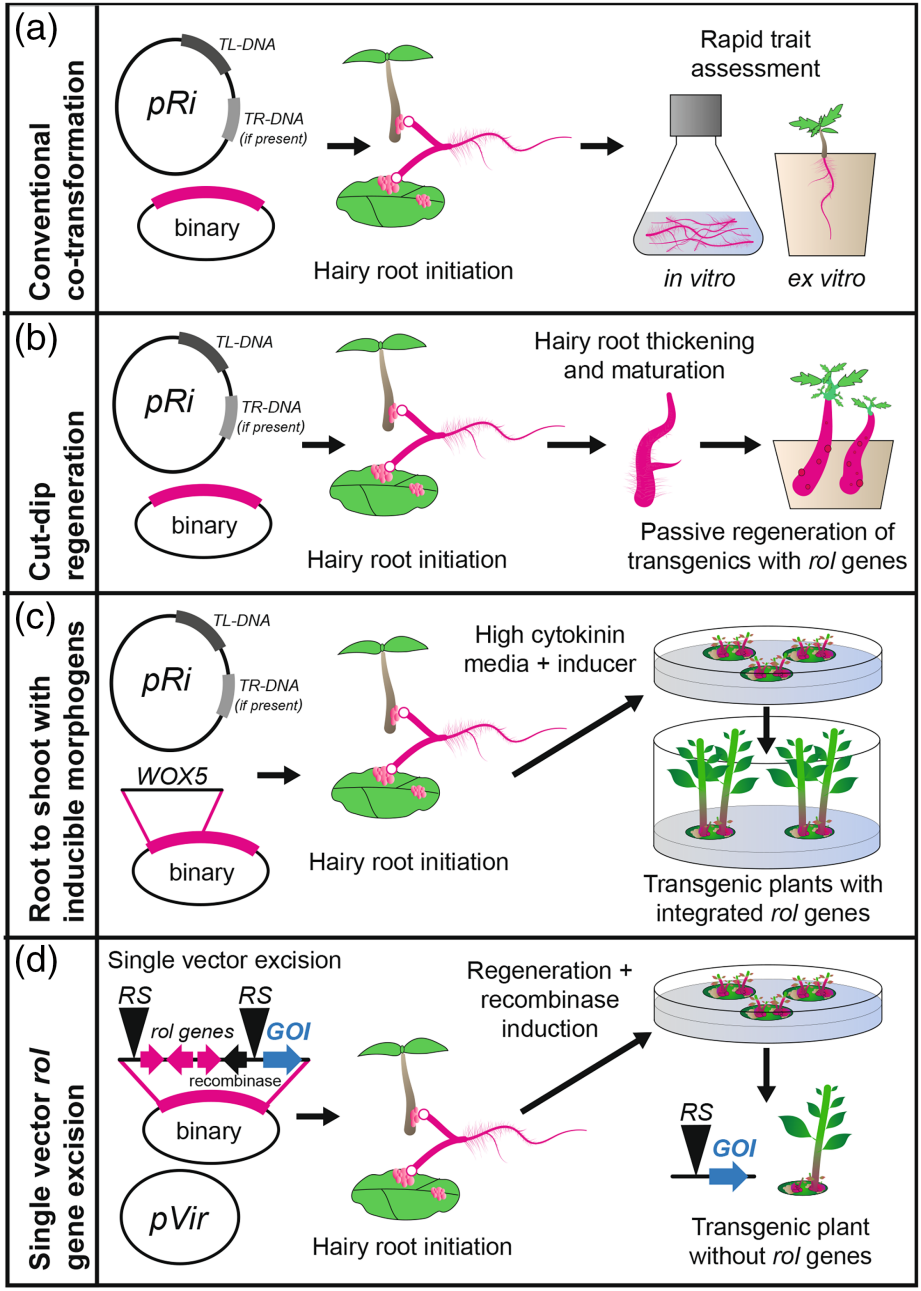
Hairy root transformation systems for recalcitrant plant species. (a) Wild strains with binary vectors containing a gene of interest for rapid trait assessment in liquid culture or composite plants. (b) Wild strains with binary vectors containing a gene of interest, using natural regenerability of mature root tissues to regenerate transgenic intact plants (cut–dip–budding method). (c) Wild strains with binary vectors containing a gene of interest and inducible morphogenic genes, allowing for highly efficient shoot regeneration in vivo. (d) Single vector systems in disarmed strains delivering hairy root‐inducing *rol* genes which are excised by a recombinase during regeneration, producing shoots with only the gene of interest integrated.

Although hairy root induction using wild strains of *A. rhizogenes* has been a common tool in plant biotechnology for several decades, its use has risen in the past few years (Kiryushkin et al., 2022). This is primarily because hairy root transformation is achievable in a much wider range of species than stable *A. tumefaciens* transformation using disarmed strains, particularly in dicots (Gomes et al., 2019; Ying et al., 2023). Even some conifers are amenable to hairy root transformation, though the quality of the produced roots and amenability to continued growth in culture is often low (He et al., 2023; McAfee et al., 1993). Nonetheless, *A. rhizogenes* transformation can be used as a rapid prototyping tool in many otherwise recalcitrant crop species and is a common platform for *in planta* screening prior to investing the considerable effort required for the production of stable transgenics (Jedličková et al., 2022; Kiryushkin et al., 2022). This is especially appealing for tasks such as testing sgRNA editing efficiency, as editing rates can be quite variable (Liu et al., 2022). Hairy roots are also useful in the rapid assessment of traits of interest that can be inferred from root tissue, such as root growth traits, pathology phenotypes, some types of secondary compound metabolism, nitrogen‐fixation symbiosis, and secondary growth (Figure 3a) (Gomes et al., 2019; Huang et al., 2022; Plasencia et al., 2016; Ron et al., 2014). Hairy roots are usually morphologically distinct from wild‐type adventitious roots but have conserved expression of genes identifying different cell files, and are thus useful for assessing developmental phenotypes (Ron et al., 2014). Composite plants, where hairy roots are formed *in vitro* attached to non‐transgenic shoot tissue, can be hardened for use in greenhouse testing of similar traits (Figure 3a).

The major limitation of hairy root culture has been that the products are root tissues, not intact plants that can be further propagated. However, several recent studies have shown that shoots can be regenerated from these transgenic hairy roots through the *in vitro* application of PGRs, though regenerants derived from hairy roots often have pleiotropic phenotypes such as increased chlorophyll content, dwarfism, altered flowering, and infertility (Godo et al., 1997; Nilsson & Olsson, 1997). Several recent innovations using these root‐then‐shoot approaches are expanding the ability to generate transgenic or edited products in difficult species (Figure 3).

The first method, first published in 2023 and supported by multiple subsequent studies, leverages the natural regenerability of mature root tissues in some species. Termed the “cut–dip–budding” inoculation approach, these methods build upon the established methodologies of composite plant hairy root production (Cao, Xie, Song, Lu, et al., 2023). In this approach, greenhouse‐derived plant materials are inoculated with (usually plate grown) *A. rhizogenes* carrying a binary vector and placed into vermiculite or similar media to allow hairy roots to form (Figure 3b). Positive roots are allowed to mature and are subsequently divided into explants and placed again in vermiculite (Cao, Xie, Song, Lu, et al., 2023; Cao, Xie, Song, Zhao, et al., 2023). Without the exogenous supply of PGRs, passive shoot regeneration from these segments can be accomplished (Figure 3b). This method has been successfully used in several species, including *Taraxacum kok‐saghyz*, *Coronilla varia*, sweet potato, several woody plant species including *Ailanthus altissima*, *Aralia elata*, and *Clerodendrum chinense*, and several succulent species (*Kalanchoe*, *Crassula*, and *Sansevieria*) (Cao, Xie, Song, Lu, et al., 2023; Cao, Xie, Song, Zhao, et al., 2023; Lu et al., 2024). For many of these species, gene editing was also demonstrated using this approach, often using *phytoene desaturase* bleaching mutations as a visual marker. This highlights that naturally regenerative tissues, in conjunction with genes from *A. rhizogenes*, can be leveraged for the production of transgenic or edited plants without tissue culture.

For plants without these natural regeneration budding pathways, tissue culture remains an option while leveraging the efficiency of *A. rhizogenes‐*mediated transformation. In this configuration, hairy roots are generated either from composite plants with attached non‐transgenic shoots or via detached explants, which are then divided and placed on high cytokinin containing media until shoots differentiate. This approach was recently shown to work across a wide range of citrus genotypes, as well as in kiwifruit (Li et al., 2024; Ramasamy et al., 2023). In the case of citrus, most of the regenerants were phenotypically normal due to silencing of the integrated *rol* genes during regeneration. However, the use of empty vector controls with this approach is essential due to the strong effects the *rol* genes can have on some events on plant development (Ramasamy et al., 2023). In addition to PGRs, one recent study not only combined the same approach but also added inducible morphogenic genes such as *WUSCHEL*, *WUSCHEL‐RELATED HOMEOBOX5 (WOX5)*, and *BABY BOOM* (Liu et al., 2024). The addition of *WOX5*‐inducible expression during both callus induction and shoot induction increased regeneration in apple from ~2% to 20% efficiency among cultured hairy root explants (Figure 3c). This same approach also worked in kiwifruit.

All the earlier innovations in regeneration systems used wild‐type Ri plasmid carrying *Agrobacterium*, with introduced binary vectors in a co‐transformation framework. Although there have been some older reports of using Ri T‐DNA encoded genes as morphogens to improve transformation, they have been quite limited and were mostly used to study the function of *rol* genes (Aoki & Syōno, 1999; Stieger et al., 2004). Among the most complicated of these transformation systems was developed in the early 2000s and included single complex T‐DNAs which combined hairy root genes, an inducible *ipt* cassette to improve shoot formation, and a recombinase excision system (Figure 3d) (Ebinuma et al., 2004; Ebinuma & Komamine, 2001). Given the recent interest in morphogens and the evolution of advanced cloning technology such as GA*A*NTRY as discussed earlier, the use of *rol* genes as parts of complex, controlled expression and excision systems in both old and new disarmed strains has tremendous promise. Combination and innovation of these strategies (particularly if the resulting events eliminate *rol* genes from the genome via crossing or excision), emphasizing *ex vitro* or entirely tissue culture‐free approaches could overcome barriers and improve transformation outputs in challenging species.

## THE PROSPECT OF HIGHLY ENGINEERED, “SUPER‐STRAINS” OF *AGROBACTERIUM*

Several developments are converging that will enable the creation of new, highly engineered *Agrobacterium* strains that can increase the efficiency and host range for plant transformation and regeneration. The combination of genomic databases, tools for targeted manipulation of *Agrobacterium*, and growing insights into genes and pathways that stimulate plant transformation and regeneration provide many new options for improving transformation systems. Major advances may be obtained through the rapid generation of new disarmed strains from a wide diversity of wild isolates, mitigation of host defense responses through multiple mechanisms, and use of directed evolution to optimize component function. Advances in methods for efficiently transferring and stacking transformation promoting genes of many kinds, into both new and old strains, should elevate host range and virulence (Figure 2d). All of these innovations are likely to lead to enhanced transgenic plant recovery, especially for recalcitrant and woody species. It appears that we have entered a new golden age of *Agrobacterium* biology and engineering.

## Data Availability

Data sharing not applicable to this article as no datasets were generated or analysed during the current study.
